# BCG vaccination: historical role, modern applications, and future perspectives in tuberculosis and beyond

**DOI:** 10.3389/fped.2025.1603732

**Published:** 2025-07-31

**Authors:** Anna Starshinova, Igor Kudryavtsev, Artem Rubinstein, Irina Dovgalyuk, Anastasia Kulpina, Leonid P. Churilov, Dmitry Kudlay

**Affiliations:** ^1^ Department of Mathematics and Computer Science, Saint Petersburg State University, St. Petersburg, Russia; ^2^ Medical Department, Saint Petersburg State University, St. Petersburg, Russia; ^3^ Medical Department, Almazov National Medical Research Centre, Saint-Petersburg, Russia; ^4^ Department of Immunology, Institution of Experimental Medicine, St. Petersburg, Russia; ^5^ Phthisiopulmonology Department, Research Institute of Phthisiopulmonology, St. Petersburg, Russia; ^6^ Department of Pharmacology, Institute of Pharmacy, I.M. Sechenov First Moscow State Medical University, Moscow, Russia; ^7^ Laboratory of Personalized Medicine and Molecular Immunology, Institute of Immunology FMBA of Russia, Moscow, Russia; ^8^ Department of Pharmacognosy and Industrial Pharmacy, Faculty of Fundamental Medicine, Lomonosov Moscow State University, Moscow, Russia; ^9^ Laboratory of Comparative Sensory Physiology, Sechenov Institute of Evolutionary Physiology and Biochemistry of the Russian Academy of Sciences, St. Petersburg, Russia

**Keywords:** BCG vaccine, immune response, COVID-19, laboratory diagnostics, tuberculosis, preventive therapy

## Abstract

Tuberculosis (TB) remains a fatal disease primarily transmitted through airborne droplets, with children who are the most susceptible, particularly in the areas with poor tuberculosis control. The BCG vaccine, developed by Albert Calmette and Camille Guérin, has a history spanning a century. This vaccine has been implemented in numerous countries, significantly reducing child mortality in regions heavily affected by TB. In this review, we aim to revisit the vaccine's development and rollout, while also highlighting its current attributes and the successful application in the Russian Federation, where 90% of newborns receive the anti-tuberculosis vaccination. Due to that practice, only a few isolated cases of young children with generalized tuberculosis (about five to seven annually) are observed in Russia. Research on the BCG vaccine is ongoing, revealing significant genetic alterations in BCG strains that have evolved from the original variant. These genetic differences may contribute to variations in vaccine efficacy, making screening important to predict effectiveness. The BCG vaccine can initiate a localized mucosal immune response, offering, besides the anti-TB effect, some protection against infections involving mucous membranes, including salmonellosis, HIV, and acute viral respiratory infections. It is essential to investigate the role of BCG in various applications; however, this exploration should not detract from its main protective benefits against tuberculosis (TB). Future studies may provide evidence of the vaccine's safety and efficacy to support its use beyond TB prevention. While BCG vaccination does not lower the risk of infection with *Mycobacterium tuberculosis*, it does prevent the progression to the most severe clinical manifestations (such as miliary TB and tuberculous meningitis) caused by hematogenous spread of *M.tuberculosis*. The challenge of protecting HIV-infected children from TB remains urgent, especially in regions burdened with drug-resistant TB, highlighting the need for robust protective measures.

## 1 Introduction

Tuberculosis (TB) remains one of the leading infectious diseases responsible for a significant number of deaths worldwide. The World Health Organization (WHO) reported that in 2023, approximately 10.8 million new TB cases were identified, marking a 3.5% rise from the 10.3 million cases recorded in 2021. Children are among the most at-risk populations, particularly in regions or countries with a high TB prevalence. In 2018, the inability to provide timely and accurate diagnoses for over 600,000 children resulted in the tragic deaths of 200,000 children of various ages. By 2020, more than 226,000 children under the age of 15 had succumbed to the disease, and recent statistics indicate that around 25,000 children contracted tuberculosis from patients with multidrug-resistant tuberculosis (1).

**Figure 1:**
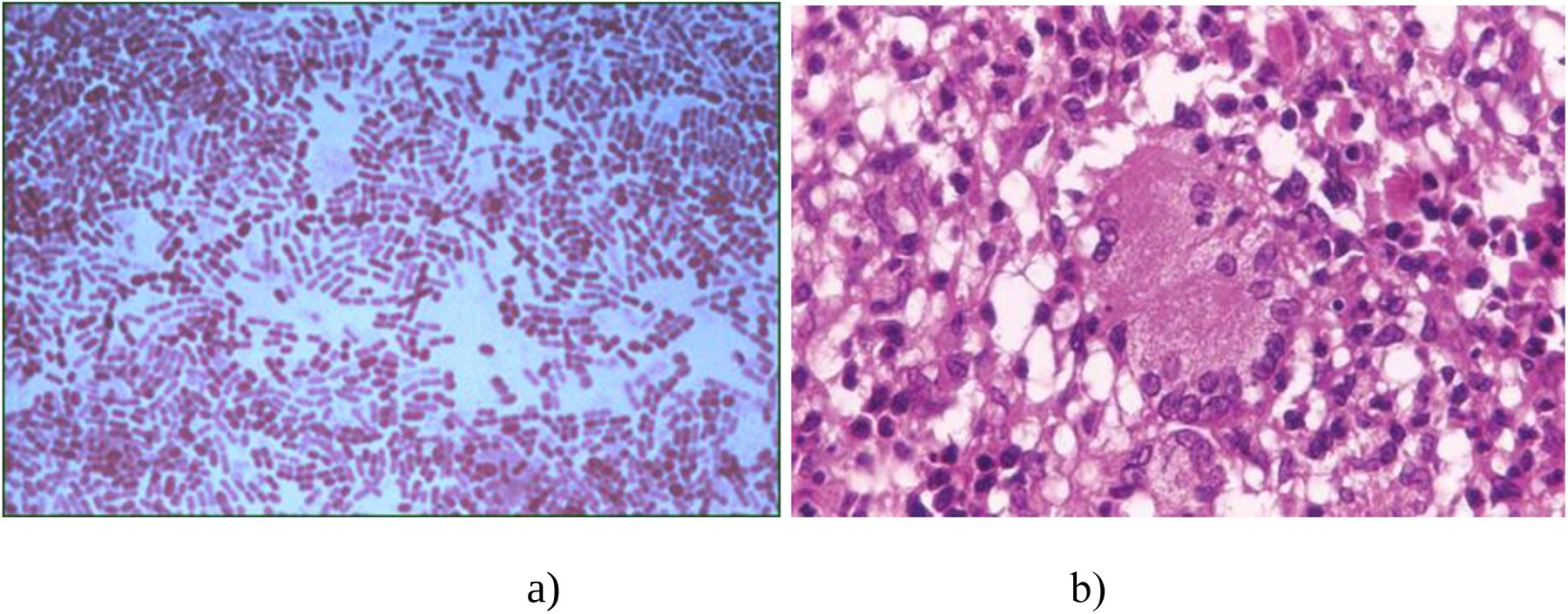
Diagnosis of BCG infection according to histological findings. **(a)** Gram staining of mycobacteria; **(b)** The microscopic structure of a tuberculosis granuloma.

Vaccination efforts have achieved significant progress, leading to a dramatic decline in the incidence of many diseases such as measles, diphtheria, tetanus, rubella, epidemic parotitis, and hepatitis B, all of which are now limited to only a few isolated cases. Polio has been eradicated in most regions, e.g., Russia maintaining its polio-free status since 2002 as part of the European Region. The USA became the first region in the world to be declared free of endemic measles on September 26, 2016. Furthermore, smallpox was declared globally eradicated in 1980.

Immunoprevention of infectious diseases encompasses a range of individual and mass strategies aimed at preventing disease onset, controlling pathogen spread, reducing infection severity, and eradicating particularly hazardous infectious diseases. At present, the implementation of early diagnostic and preventive measures for tuberculosis infection is of particular importance, as it directly correlates with the prevention of active tuberculosis transmission. It is well established that a single individual with active tuberculosis can infect up to 30–50 people per day, especially through close contact. Additionally, it focuses on enhancing the immune response to specific pathogens (2). Under special circumstances, individual immunization may also serve a therapeutic role. Mass immunization is employed during epidemics when there is a risk of widespread infectious diseases. The term “*vaccine*” is derived from the Latin word vacca, meaning cow. A vaccine is a medical or veterinary preparation designed to create active immunity to infectious diseases. Vaccines are developed using attenuated or inactivated microorganisms, byproducts of their biological activity, or antigens produced through genetic engineering or chemical methods (3). Live vaccines are created using attenuated strains of microorganisms that retain consistent avirulence (non-pathogenic properties). Once administered, these strains replicate within the host cells, leading to a controlled vaccine-induced infection. Examples of live vaccines include those for rubella, measles, polio, tuberculosis, and mumps. Because antigens of live vaccines are produced within host cells by the persisting pathogens, they are processed and presented mostly via intracellular (although also via extracellular, phagocytosis-associated) routes, in the context of both MHC Class I and Class II proteins. Hence, this type of vaccine is able to induce active cellular, as well as humoral adaptive immune responses, making it the most effective. The history of immunoprophylaxis began with a milestone achieved by English physician Edward Jenner. In 1796, he vaccinated the eight-year-old son of his gardener using live cowpox virus. Jenner proposed that material derived from cowpox lesions could be used for immunization, and individuals who received this inoculation were protected from smallpox. However, there is historical evidence that Jenner's experiment was preceded by analogous successful procedure performed on three children in 1791 by a German schoolteacher, Peter Plett, who has reported his experience at the University of Kiel, but the medical professors severely criticized «*the amateur without M.D. Degree*», so first publication of Plett's results was postponed until 1802. In 1880, Louis Pasteur developed a vaccine against anthrax, followed later by vaccines against cholera and rabies. Then, in 1921, Albert Calmette and Camille Guérin announced the development of a vaccine against tuberculosis (4). Vaccines can be derived from pathogens and their byproducts. They are classified into two main types: Live vaccines, which contain attenuated pathogens, and non-live (inactivated) vaccines, which do not contain any live microorganisms (1).

## 2 History of the BCG vaccine

The BCG vaccine (Bacillus Calmette–Guérin, BCG) is prepared from a strain of attenuated *Mycobacterium bovis* BCG. This strain is produced in an artificial environment and has low virulence in humans. The vaccine was developed due to the collaborative efforts of two French scientists: navy physician and bacteriologist Léon Charles Albert Calmette, and veterinarian and immunologist Jean-Marie Camille Guérin. Being a student, Camille Guérin began working as an assistant to the renowned pathologist Edmond Nocard (4). In 1902, Nocard isolated a culture of *M. bovis* from a cow suffering from tuberculosis. He authored a monograph: *La Tuberculose Bovine: ses Dangers, ses Rapports avec la Tuberculose Humaine*. In 1912, Nocard and Norwegian veterinarian Christian Feyer Andvord (who coined the idea to use ox bile to weaken pathogens), after 96 serial inoculations, succeeded in obtaining an attenuated culture of *M. bovis* using a nutrient medium composed of bile, potato, and glycerol (4) (Figure 1).

After considerable delay caused by The Great War, in 1919, Albert Calmette established a working group at the Pasteur Institute in Paris to develop a tuberculosis vaccine. By that same year, researchers conducted 230 serial passages, demonstrating changes in the morphological and cultural characteristics of M. bovis, as well as a reduction in its virulence in experimental models (5). The safety and effectiveness of the tuberculosis vaccine in veterinary medicine were confirmed at the experimental farm in Fécamp in 1921. That year, scientists announced that the BCG vaccine was ready for practical use, marking the beginning of mass vaccination efforts. On July 18, 1921, pediatricians Benjamin Weill-Hallé and Raymond Turpin initiated vaccinations for newborns at the Hôpital de la Charité in Paris (6). The first infant to receive the vaccine (initially designed for veterinary practice) was born to a mother who had died from tuberculosis just a few hours after childbirth. The child was administered the vaccine orally on the 3rd, 4th, and 7th days of life.

After six months of monitoring, it was determined that the child exhibited no signs of illness, despite being in close contact with the infected mother. From 1921–1924, tuberculosis vaccination was gradually extended to include other newborns at the Hôpital de la Charité. In 1921, Weill-Hallé introduced oral administration of BCG at the hospital (7, 8). This method was further developed by Boquet and Nègre, who continued the use of oral BCG emulsions. The effectiveness of this delivery pathway depends on the status of the gastrointestinal tract, with post-vaccination allergic reactions observed in 30% of cases (8).

**Figure 2:**
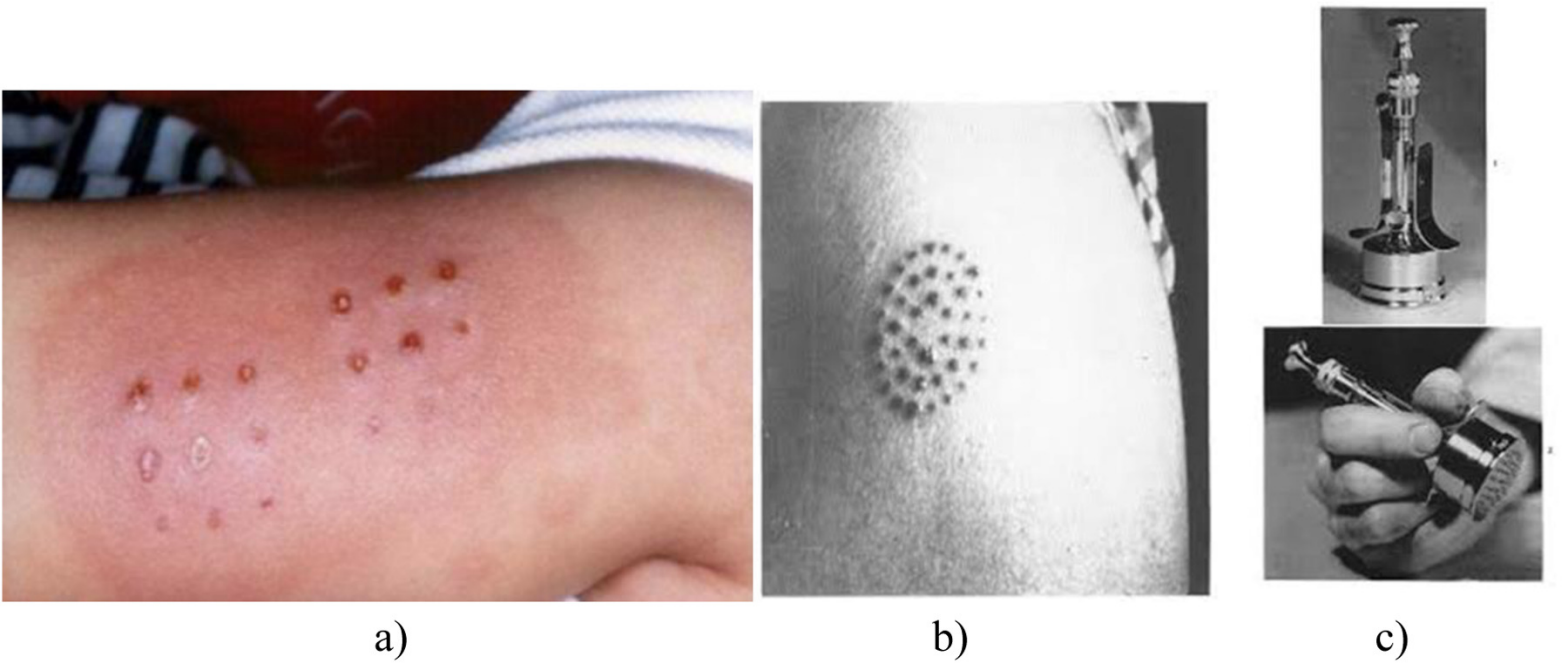
Multiple jabs during intradermal administration of BCG vaccine (10). **(a)**—multiple vaccination marks; **(b)**—the multiple injection technique; **(c)**—multiple injection vaccination machine).

Starting in 1924, BCG vaccination was implemented in French healthcare dispensaries. Between 1924 and 1925, the vaccination campaign expanded to Madagascar and Indochina. In 1925, Canada established the Tuberculosis and BCG Research Committee under the Medical Research Council, aimed at evaluating the vaccine's effectiveness in both humans and animals. That same year, F.A. Baudouin began clinical trials of the vaccine (9). By 1927, Calmette published a study detailing the vaccination outcomes of 21,200 newborns, providing compelling evidence of the BCG vaccine's efficacy. The same year, Swedish pediatrician Karl Nöslund also demonstrated, using large statistical data, that BCG vaccination significantly reduced infant mortality, which contributed to its public acceptance across Scandinavia. In 1928, the League of Nations officially recognized the vaccine. Also in 1927, Luis Sayé in Barcelona, Arvid Wallgren in Gothenburg, and Johannes Heimbeck in Oslo were the first to administer BCG intradermally using the multiple-injection technique (10) (Figure 2).

The method was modified and applied in France and further in the USA using a multiple injection apparatus developed by Konrad Birkhaug (1927) (11).

### 2.1 The Lübeck tragedy

**Figure 3:**
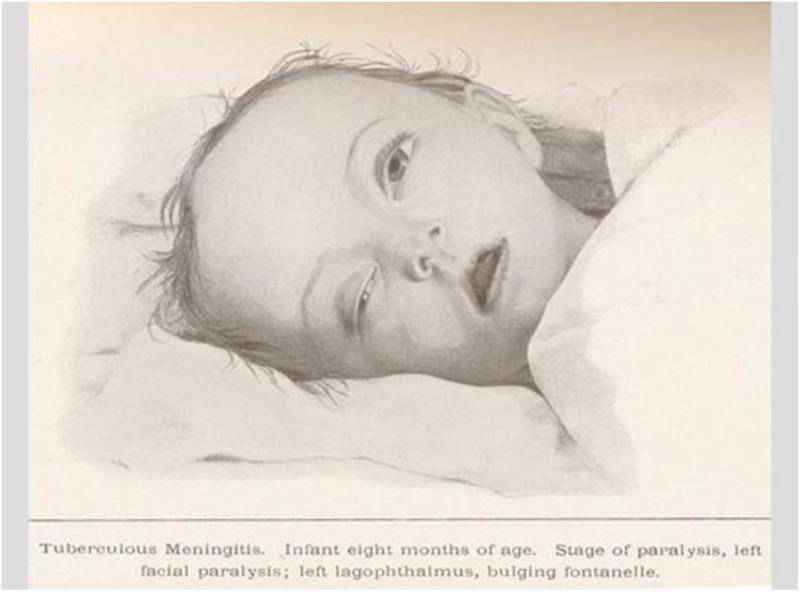
Child with generalized tuberculosis after BCG vaccination in the Lübeck tragedy (10).

Probably due to differences in the versions of the vaccine used at different times and in various places, and even more so due to the influence of subjective and random factors, as well as non-scientific circumstances influencing public opinion, the social acceptance of BCG in many parts of the world was greatly delayed or did not occur at all. In 1930, the Lübeck tragedy broke out (Figure 3). Four to six weeks after BCG vaccination, 72 out of 251 newborns died within a year from generalized tuberculosis (10, 12).

A total of 131 children developed clinical tuberculosis, which was ultimately treated successfully. As a result, the German government filed a lawsuit against the Pasteur Institute. An investigation was launched in late 1931, led by Professor Bruno Lange of the Robert Koch Institute in Berlin and Professor Ludwig Lange of the German Ministry of Health (10). After 20 months of thorough inquiry, the BCG vaccine was exonerated, while the Lübeck laboratory was found responsible for contaminating vaccine batches with virulent strains of *Mycobacterium tuberculosis*. Two medical professionals were convicted and sentenced to prison. Additionally, in August 1930, at the International Union Against Tuberculosis congress in Oslo, Calmette publicly defended the BCG vaccine, reaffirming its safety and efficacy. However, the tragedy in Lübeck delayed the acceptance of BCG by the German healthcare system. Moreover, during that period, the BCG vaccine was not adopted for use either in Great Britain or in the USA. Its introduction into medical practice in Britain was prevented by the position of the authoritative microbiologist M. Greenwood, who in 1928 sharply criticized the methodology of Calmette's statistical calculations in the high-impact “British Medical Journal”. In the USA, S.A. Petroff and co-authors at the largest phthisiology center, the Trudeau Sanatorium, analyzing in 1929 a sample sent by A. Calmette, found virulent *Mycobacterium tuberculosis* in it (11), which practically buried the prospects for the rapid introduction of the new product overseas.

**Figure 4:**
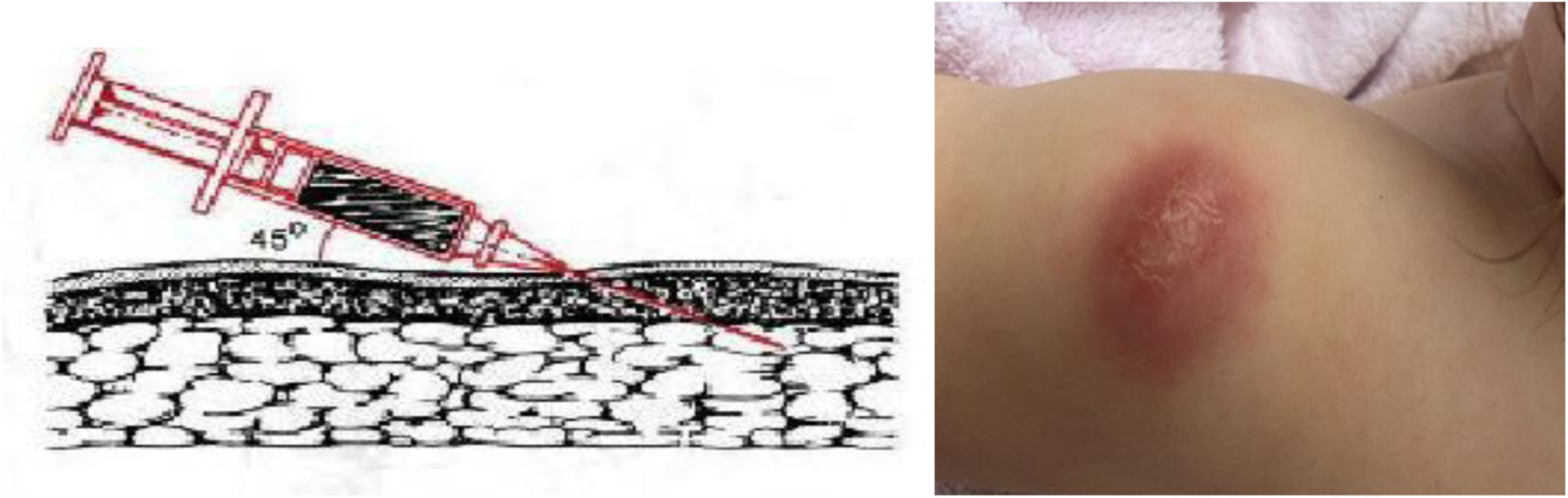
BCG vaccine subcutaneous injection technique and cold abscess formation.

Meanwhile, B. Weill-Hallé (1930) applied a subcutaneous method of BCG administration (Figure 4). Unfortunately, there were many complications, including cold abscesses that persisted for a long time (12, 13).

### 2.2 Percutaneous administration of BCG emulsion

Roy Rosenthal (1939) applied the method of multiple needle pricks of the skin in place of a drop of BCG emulsion and B. Weill-Hallé (1939) applied the scarification method (14). A drop of emulsion is scratched through the entire epidermis followed by a compress made of gauze soaked in vaccine. However, this method has not been widely used (13).

## 3 Use of BCG vaccine in the world and in Russia

Historically, the earliest recognition and pioneering use of BCG for mass vaccination occurred in its country of origin—France—as well as in several Scandinavian countries and the USSR (see below). However, prior to World War II, BCG vaccination was not mandatory in any of these countries. In 1946, the Danish Red Cross coordinated BCG vaccination programs in Poland, Austria, Hungary, and Yugoslavia (15). Norway mandated BCG vaccination for individuals with negative tuberculin skin tests in 1947, followed by France in 1950, which introduced compulsory vaccination. That same year, the Soviet Union also implemented mandatory BCG vaccination for all newborns. In 1974, the BCG vaccine was incorporated in the Expanded Programme on Immunization (EPI) by the United Nations International Children's Emergency Fund (UNICEF). In the United States, BCG vaccination was reserved for individuals at high risk of tuberculosis exposure. According to World Health Organization (WHO) guidelines, BCG vaccination remains a cornerstone of global TB prevention strategies. Currently, BCG vaccination is mandatory in 64 countries and recommended in an additional 118 countries and territories. Following World War II, the issue of mass BCG vaccination was reconsidered in both the United Kingdom and the United States. However, findings from cohort studies differed significantly between the two countries: high effectiveness was reported in the UK, while no significant benefit was observed in the US. This discrepancy is thought to be attributable to methodological differences—such as the use of various BCG strains, stricter age matching in the British cohort, and limited control in the American studies due to populations residing in regions with frequent exposure to animals carrying *Mycobacteria* (12). Notably, neither the United States nor the Netherlands has ever implemented a universal BCG vaccination program. A meta-analysis published in 1995 demonstrated that BCG vaccination in neonates and infants reduces the risk of developing tuberculosis by an average of more than 50% (16). A robust and protective immune response following BCG vaccination has been observed across diverse populations, study designs, and TB manifestations.

In 1925, Albert Calmette, a disciple of Ilya Metchnikoff, provided a prototype BCG strain to another of Metchnikoff's students—Soviet Professor Leo A. Tarasevich in Moscow—where it was designated as BCG-1. The first BCG vaccinations of newborns in tuberculosis-endemic regions of the Soviet Union began in 1928 (5). A national policy mandating BCG vaccination for newborns in urban maternity hospitals was introduced in 1942 (Order of the People's Commissariat of Health No. 448, dated August 31, 1942). By 1953, BCG vaccination coverage had expanded to include newborns in rural areas, as well as primary vaccination and revaccination of all children of preschool age and schoolchildren not infected with *Mycobacterium tuberculosis* (in accordance with Decree of the USSR Council of Ministers No. 3989, dated October 25, 1948, and Orders of the USSR Ministry of Health No. 676 of November 12, 1948, and No. 384 of July 3, 1952). BCG vaccination has been continuously administered in the USSR and subsequently in the Russian Federation since 1953.

**Figure 5:**
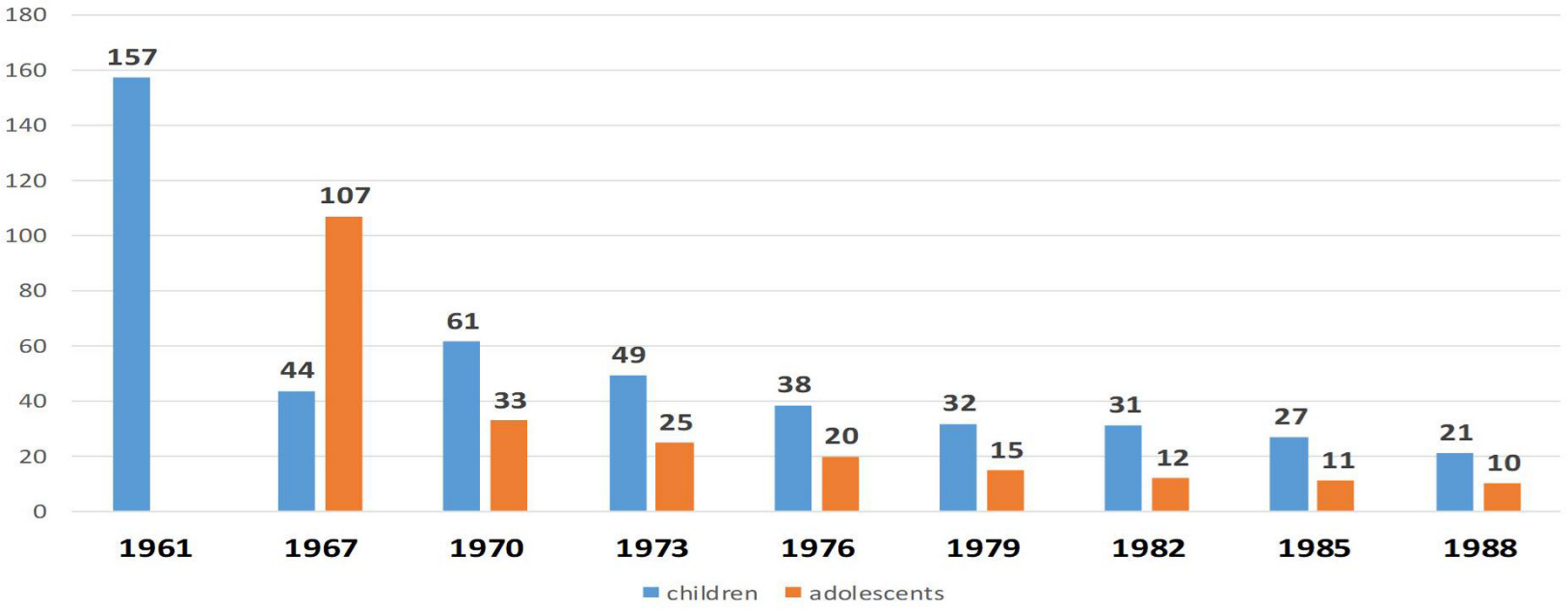
The dynamics of morbidity among children and adolescents with tuberculosis in the USSR after the introduction of intradermal immunization with BCG vaccine (cases per 100,000 population)[y, years; x, children (blue) and adolescents (orange)].

Until 1962, oral administration was the predominant method for newborns, with percutaneous administration used less frequently. Since 1962, the intradermal route has been adopted as the standard one due to its superior immunogenicity. In the Russian Federation, a single BCG revaccination is administered at age seven for children with a negative Mantoux test with 2 TE PPD-L. Re-vaccination at age 14 was discontinued in Russia in 2014. Trends in tuberculosis incidence among children and adolescents in the USSR following the introduction of intradermal BCG immunization (per 100,000 population) are illustrated in Figure 5.

**Figure 6:**
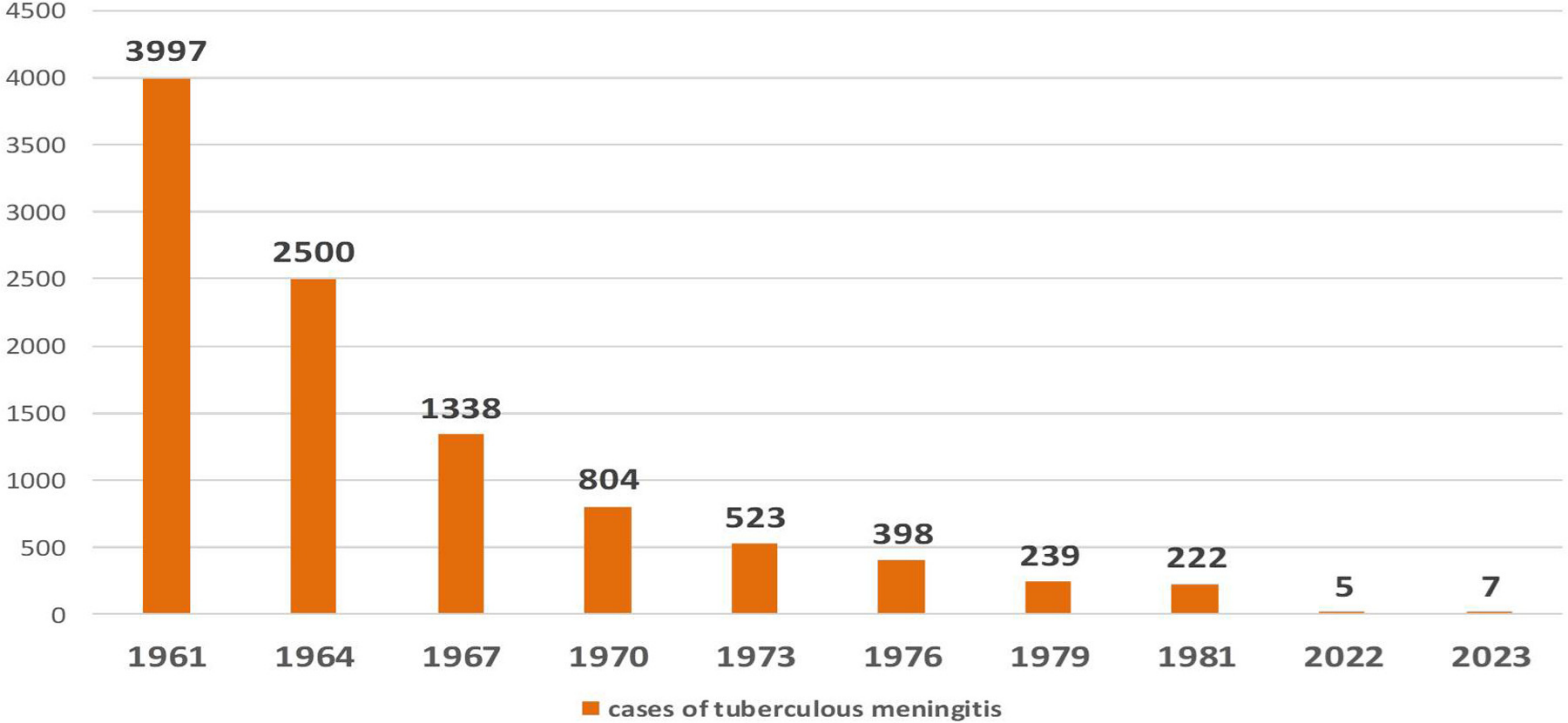
Dynamics of the letal cases of, tuberculous meningitis after the introduction of mass BCG vaccination in the USSR. (y, years; x, number of children with meningitis).

Since 1961, a steady decline in tuberculosis incidence has been observed among both children and adolescents throughout the USSR. The implementation of BCG vaccination in Russia has contributed to several notable public health achievements, including:

- The elimination of fatal tuberculosis cases in children during periods of high TB incidence, particularly the eradication in young children with tuberculous meningitis and miliary tuberculosis (Figure 5);
- Stable TB prevalence rates, with no observed increase in complicated or disseminated forms; A predominance of lymph node involvement over systemic disease manifestations.

The epidemiological trends of tuberculous meningitis following the introduction of mass BCG vaccination in the USSR are depicted in Figure 6.

Currently, tuberculosis vaccination coverage among newborns in the Russian Federation reaches 90%–95% (2). National surveillance data from the past decade indicate that only five to seven cases of tuberculous meningitis are reported annually. In 2023, a total of ten confirmed cases were reported (17).

## 4 BCG vaccine strains

**Figure 7:**
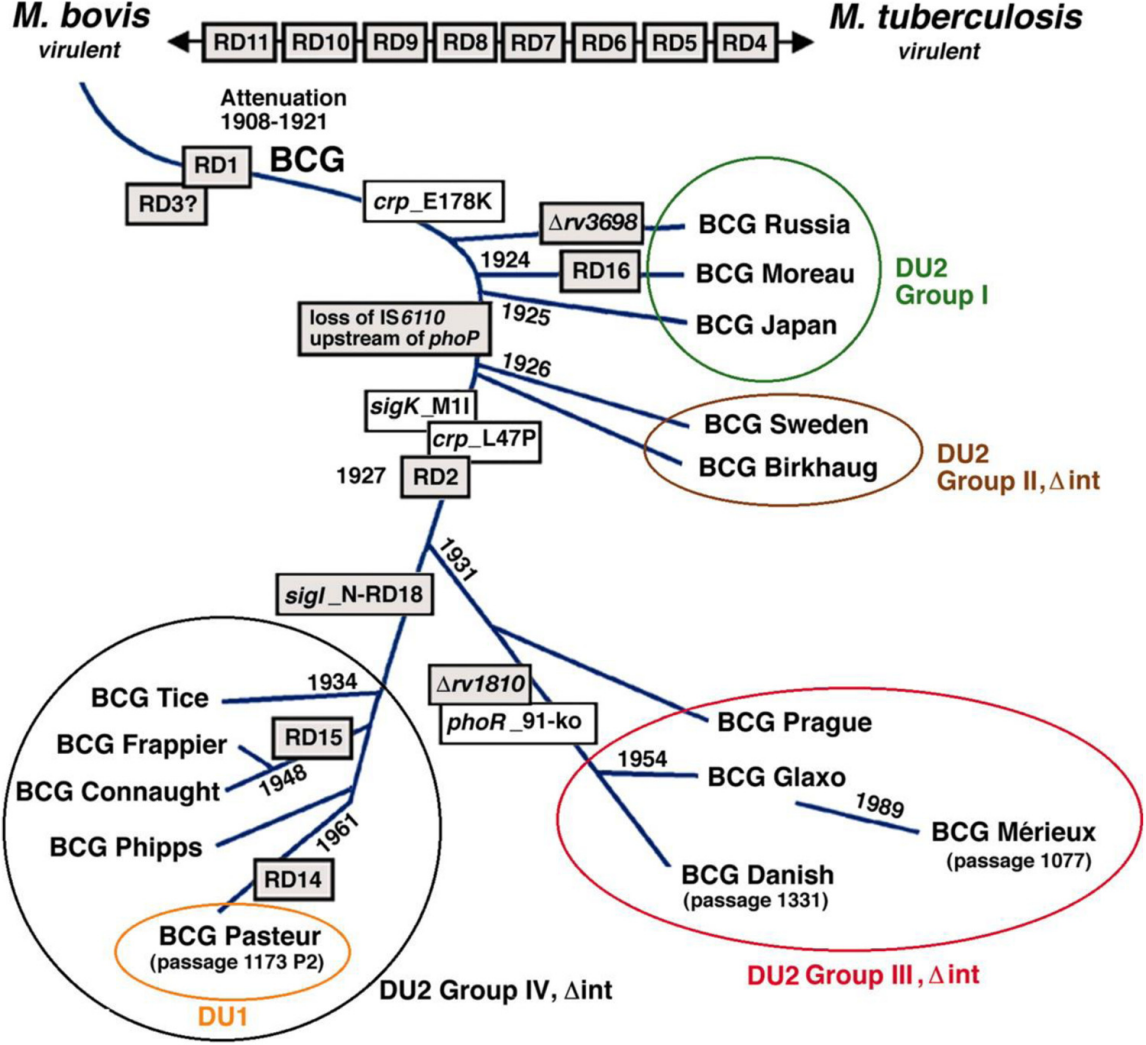
Phylogeny of BCG sub-strains with shwing genetic deletions in the different vaccine sub-strains over time (18).

Since 2004, the following strains have accounted for 90% of BCG vaccinations worldwide (18, 19) (Figure 7):

- Pasteur 1,173 P2 (France);
- Danish 1,331 (Denmark);
- Glaxo 1,077 (Danish derivative)
- Tokyo 172-1 (Japan);
- BCG-1 (Russia).

According to the most recent update of the BCG World Atlas (2020), the most widely used BCG vaccine strains globally are Danish 1,331 (16.6%), Pasteur 1173P2 (9.2%), and Tokyo 172 (7.3%) (19). The present study aimed to analyze the complete genome sequences of two of the most widely used BCG strains: the WHO-recommended reference strain (Danish 1,331) and the Pasteur 1173P2 strain used in Iran. A total of 4,060 genes were identified in Pasteur 1173P2 and 4,037 genes in Danish 1,331 using a comparative annotation framework. Among them, 4,006 coding sequences (CDSs) and 50 tRNA genes were found in Pasteur 1173P2, while Danish 1,331 contained 3,982 CDSs and 51 tRNA genes. Both strains contain three rRNA genes (5S, 16S, and 23S) and a single tmRNA gene (ssrA) (19).

When compared to the *Mycobacterium tuberculosis* H37Rv reference genome, both strains were found to contain 58 PPE genes and 31 PE family genes. Additionally, 58 PE_PGRS subfamily genes were identified in Pasteur 1173P2, compared to 59 in Danish 1,331. Notably, specific genomic deletions and insertions—referred to as regions of difference (RDs)—that distinguish BCG sub-strains were identified: RD14 and N-RD18 were present in Pasteur 1173P2 but absent in Danish 1,331.Conversely, a DU1-like region spanning 14,577 base pairs was found in the Danish 1,331 strain.

There is growing evidence that genetic drift among BCG strains has led to significant variability in their immunogenicity and protective efficacy (16). Identifying these genetic differences is essential for understanding clinical heterogeneity and informing future vaccine development.

### 4.1 Genetic evolution and virulence of BCG sub-strains

The phylogenetic lineage of modern BCG sub-strains can be traced back to the original *Mycobacterium bovis* BCG strain developed at the Pasteur Institute. These sub-strains have diverged over time, with some exhibiting abrupt genetic changes after 1927, including gene deletions and alterations in biochemical phenotypes.

Historical and molecular evidence supports the hypothesis that BCG strains produced at the Pasteur Institute in the late 1920s underwent attenuation of virulence (19). Today, BCG vaccine strains vary in their ability to induce immune responses. Multiple studies have demonstrated significant differences in both the magnitude and quality of neonatal immune responses elicited by various strains (20). In particular, BCG-Danish and BCG-Japan have been shown to induce higher frequencies of polyfunctional cytotoxic T lymphocytes and elevated production of Th1-type cytokines.

Despite its widespread use, the full protective potential of the BCG vaccine has not yet been fully realized (20). Although it confers strong protection against severe forms of tuberculosis in children—such as miliary TB and tuberculous meningitis (16)—its efficacy in preventing adult pulmonary TB, particularly in high-burden settings, remains limited.

Given that approximately one-quarter of the global population is infected with *Mycobacterium tuberculosis* (Mtb), there is an urgent need for novel vaccines designed for both pre-exposure (preventive) and post-exposure (therapeutic) use. Current clinical trials are evaluating endpoints such as prevention of infection, disease progression, and relapse. Most vaccine candidates target T cell–mediated immune responses, particularly CD4^+^ and CD8^+^ T lymphocytes, which are essential for controlling Mtb infection.

## 5 Next-generation TB vaccines

Despite widespread use, the full protective potential of the BCG vaccine has yet to be fully realized (20). While it provides strong protection against severe forms tuberculosis in Children, such as miliary tuberculosis and tuberculous meningitis (16), BCG is less effective in preventing adult pulmonary TB, particularly in endemic settings.

Given that approximately one-quarter of the global population is infected with *M. tuberculosis* (Mtb), there is a pressing need for new vaccines designed for both pre-exposure (preventive) and post-exposure (therapeutic) applications. Clinical trial endpoints currently under investigation include prevention of infection, disease progression, and relapse. TB vaccine development primarily targets T-cell–mediated immune responses, particularly CD4^+^ and CD8^+^ T lymphocytes, which are key to containing Mtb infection.

Current vaccine strategies include:

1. Whole-Cell Vaccines
  • Recombinant BCG (rBCG) strains engineered to express foreign antigens (e.g., *ESAT-6* encoded in RD1 regions);
  • Incorporation of the Listeria monocytogenes hemolysin gene to enhance phagosomal escape;
  • Addition of immunodominant antigens such as Ag85A, although some constructs inadvertently confer antibiotic resistance (21, 22).
2. Subunit Vaccines
  • Fusion proteins of mycobacterial antigens combined with Th1-stimulating adjuvants (e.g., monophosphoryl lipid A).
  • Examples include Mtb72F, a fusion of two immunodominant antigens;
  • Other candidates target Ag85, ESAT-6, CFP-10, or heat shock proteins like DnaK.
3. DNA Vaccines
  • Plasmid-based constructs encoding Mtb antigens;
  • Capable of inducing cytotoxic T lymphocyte (CTL) responses and long-lasting immunity;
  • Some vaccines combine antigens of 6 kDa and 32 kDa with lipophilic adjuvants.

A promising candidate, H4:IC31, underwent Phase II clinical trials in South Africa in 2018. It was utilized a viral vector presenting the Ag85 antigen via the Modified Vaccinia Ankara (MVA85A) platform. However, its efficacy was inferior to that of BCG (23).

Two other genetically modified live vaccines, VPM1002 and MTBVAC, are currently in various phases of clinical development.

### 5.1 Russian contributions: GamTBvac and GamLTBvac

The Gamaleya National Research Center for Epidemiology and Microbiology (Russia) has developed two novel TB vaccines: the prophylactic GamTBvac and the therapeutic GamLTBvac. Currently in Phase III clinical trials, GamTBvac is designed as a booster vaccine containing the Ag85A and ESAT 6/CFP 10 antigens, formulated with a CpG oligodeoxynucleotide (ODN) adjuvant. It is considered the most advanced subunit TB vaccine currently under evaluation in the Russian Federation. Phase I/II trial data confirmed the vaccine's safety and immunogenicity, with durable T cell responses observed in 94%–98% of participants (24). Unlike BCG—which is primarily effective in children—GamTBvac targets adolescents and adults and does not contain live mycobacteria, making it suitable for use in immunocompromised individuals.

## 6 Major types of memory T cells and broad spectrum of BCG vaccine effects

Currently, several antigen-specific memory T cell subsets are recognized, each with distinct functional characteristics and phenotypic markers (24). Initially, circulating CD4^+^ and CD8^+^ memory T cells were classified based on their homing receptor expression—CD62l and CCR7—reflecting their capacity to migrate to secondary lymphoid organs (25). This classification defined two primary subsets: central memory T cells (TCM, CD62l^+^CCR7^+^) and effector memory T cells (TEM, CD62l^−^CCR7^−^).

TCM cells exhibit high proliferative and clonal expansion potential upon antigen re exposure and secrete substantial amounts of IL 2, although they lack immediate effector functions. In contrast, TEM cells are characterized by migration-associated molecule expression, limited proliferative capacity, and abundant effector cytokine production upon activation (26).

In addition to circulating subsets, tissue resident memory T cells (TRM) have been identified in non inflamed peripheral tissues, particularly at mucosal and epithelial barriers.

These cells exhibit minimal recirculation through blood or lymphatic systems but mount rapid effector responses upon local antigen re encounter, contributing to early mucosal defense prior to systemic immunity.

Phenotypically, TRM cells can be distinguished by surface markers CD69 and CD103; however, their biology and function in humans remain incompletely understood (27). Another distinct subset, stem cell like memory T cells (TSCM), exhibit a naïve like phenotype—expressing CD45RA, CCR7, CD62l, CD27, and CD28—while also expressing memory-associated markers such as CD95, CD122, and CXCR3 (28). TSCM cells are characterized by longevity, self-renewal capacity, and high proliferative potential. Upon antigenic restimulation, they can differentiate into other memory and effector subsets, thus contributing to durable immunological memory and sustained protective responses (29). Given the critical role of TSCM cells in durable immunity, vaccine strategies—including those targeting *Mycobacterium tuberculosis*—should aim to promote their development.

Early studies on BCG-specific memory T cell phenotypes demonstrated that primary BCG vaccination in neonates induces both central and effector memory CD4^+^ T cell responses (30). IFN-*γ* and IL-2-expressing CD4^+^ T cells were initially characterized as CD45RA^−^CCR7^−^CD27^+^, consistent with effector memory phenotype. However, a substantial proportion of IFN-γ^+^ CD4^+^ and CD8^+^ T cells exhibiting a CD45RA^+^CCR7^+^CD27^+^ phenotype were also detected. At the time, these were wrong classified as central memory cells, since the TSCM subset had not yet been defined. Similar observations were made in murine *in vivo* models, where BCG vaccination led to accumulation of CD4^+^CD44^hi^CD62L^lo^ effector cells in the lungs capable of producing IFN-*γ*. Simultaneously, a population of T cells with a naïve-like phenotype also emerged (31).

Notably, adoptive transfer experiments demonstrated that BCG-specific CD44^lo^CD62L^hi^ T cells—but not their CD44^hi^CD62L^lo^ counterparts—were the key to protecting Rag^−^/^−^ mice from experimental *M. tuberculosis* infection. This finding underscores the essential role of TSCM cells in protective immunity. More recently, BCG-specific TSCM cells have also been identified in humans (32). Mpande et al. demonstrated that CD45RA^+^CCR7^+^CD27^+^ CD4^+^ TSCM cells secreting IFN-γ, TNF-α, or IL-2 were abundant in the peripheral blood of QFT-positive, HIV-negative, TB-naive individuals. These cells also expressed CD95 and CXCR3, characteristic of the TSCM phenotype, and their frequency positively correlated with the proliferative potential of BCG-specific CD4^+^ T cells measured 10 months post-vaccination. These findings suggest that BCG-induced differentiation and expansion of TSCM cells contribute to long-term immunological memory and robust recall responses against *Mycobacterium tuberculosis* (33).

Despite the emergence of TSCM, the predominant phenotype among BCG-reactive CD4^+^ T cells remains the CD45RA^−^CCR7^−^ effector memory subset. Upon BCG re-vaccination, this population further expands in peripheral blood, while increases in central memory and effector populations are less pronounced, and levels of naïve-like CD45RA^+^CCR7^+^ cells remain largely unchanged (33). Experimental evidence also supports the critical role of BCG-induced TRM cells in protective immunity. For instance, intratracheal BCG vaccination in mice significantly enhanced the formation of both CD4^+^ and CD8^+^ TRM cells in the lungs, leading to superior protection compared to subcutaneous immunization (34, 35). Additionally, adoptive transfer of pulmonary TRM cells into naive mice conferred resistance to subsequent *M. tuberculosis* infection, highlighting the importance of mucosal vaccination routes (36).

Another critical correlate of vaccine-induced protection is the generation of “polyfunctional” memory T cells capable of simultaneously producing IFN-γ, TNF-α, and IL-2 in response to antigenic stimulation (37). The role of these polyfunctional cells in protection against TB was first demonstrated by Darrah et al. in both murine models and human studies (37). These cells were present in the lungs and spleens of vaccinated mice 2–8 months post-immunization but were undetectable at 14 months (38, 39). Nonetheless, epidemiological studies in humans have shown that BCG-induced protection can persist until school age (40), and some evidence even suggests protection may last 50–60 years (41).

BCG vaccination in neonates has consistently been shown to induce polyfunctional memory T cells across diverse populations and settings (42). Interestingly, the timing of vaccination—whether administered immediately after birth or 6–10 months later—did not significantly influence the magnitude of these responses (39, 42, 43). Studies by Kagina et al. and Smith et al. found that BCG-specific polyfunctional T cells peaked in circulation 6–10 weeks post-vaccination but remained detectable up to 12 months (39, 41).

In adults, approximately 50% of the BCG-specific memory T cell pool consists of polyfunctional cells (44). Boer et al. reported that their frequency peaks around 8 weeks post-vaccination, followed by a notable decline after one year (45). Nonetheless, the development of polyfunctional memory T cells appears to be a consistent feature of BCG vaccination in both children and adults, with a minimum induction period of approximately 10 weeks (46).

Finally, evidence supports the involvement of BCG in initiating mucosal immune responses critical for protection against infections with transmucosal entry points, including tuberculosis, HIV, salmonelloses, and acute respiratory infections (47).

The BCG is a classical adjuvant, because it contains the components able to enhance the immune responses not only against target tuberculosis germ and other *Mycobacteria* (for example, those causing leprosy and Buruli ulcer), but also towards many other pathogens. It is not occasional that Jules Freund included inactivated dried *M. tuberculosis* into composition of his famous complete Freund's adjuvant broadly used since 1942 in experimental immunology (48).The type of adjuvant effect inherent in the BCG vaccine, caused by early contact of the immune system with factors modifying interactions between immunocompetent cells regardless of their clonal affiliation, has in recent years come to be referred to as “trained immunity” (49).

This phenomenon is addressed to the complex of links of the adaptive immune response. It is mediated also by the influence on the initiation of innate immunity through epigenetic mechanisms. Trained immunity as a type of adjuvant effect has its own characteristics. This phenomenon is inherent in very early and prolonged effects on the developing immune system, which is ensured by the neonatal administration of the live the BCG vaccine, essentially creating a symbiosis of the host organism and the vaccine strain of *Mycobacteria*. Due to this, epigenetic changes occur in the regulation of genome expression in myeloid progenitor cells and, in particular, in lymphoid elements of innate pools and diffuse non-encapsulated mucosa-associated lymphoid tissue. It changes the course of their differentiation, leading to greater accessibility of the genes of NOD2-dependent reactions of innate immunity for bioregulators, and reprogramming of cells for more effective production of a number of cytokines and increased expression of several Toll-like receptors. The phenomenon affects the debut phase of anti-infective protection and promotes a more active interferon response from lymphocytes (50, 51).The spectrum of documented enhancing effects of BCG vaccination on various aspects of immune protection beyond phthisiology is quite broad. It reduced the viral load and increased resistance to yellow fever, changing the cytokine profile of vaccinated individuals accordingly (52). It also proved to be an effective means of increasing antimalarial resistance in children of sub-Saharan Africa as well as in experiments on mice (53). Of special interest is use of BCG vaccine for activation of anti-neoplastic immunity. In treatment of bladder cancer, the BCG vaccine has been used locally for over 45 years. After intravesical administration of the BCG vaccine, a local immunological reaction of bladder mucosa was detected with increase in the number and activity of local immunocompetent cells (54). The comparative epidemiological data from East Germany (where BCG vaccination was mandatory between 1953 and 1991 and recommended during 1951-1952 and in 1992-1998) and West Germany (where it never was mandatory, just voluntary recommended in 1955-1998) witness for lower incidence of lymphomas and acute lymphoblastic leukemia in cohorts immunized by BCG compared to those non-immunized by this vaccine. After cancellation of mandatory BCG vaccination the incidence of lymphoid malignancies in Eastern lands of Germany tended to increase reaching the level of its Western part (55, 56).

## 7 COVID-19 and BCG vaccination

During the COVID-19 pandemic, the BCG vaccine attracted considerable attention due to accumulating evidence suggesting a correlation between BCG vaccination and reduced COVID-19 morbidity and mortality (57). Comparative analyses of COVID-19 outcomes in countries with and without universal BCG vaccination programs revealed that nations lacking such policies—such as Italy, the Netherlands, the USA, Belgium, Spain, and Sweden—experienced higher morbidity and mortality rates compared to countries with longstanding BCG vaccination practices, including former Soviet republics, Eastern European nations, South and East Asian countries, Japan, Finland, and several African states. Notably, Sweden, which ceased mandatory BCG vaccination in 1975 and did not reinstate it after 1986, reported an incidence rate approximately 4.5 times higher than that of neighboring Scandinavian countries (8,305 cases per million) and a significantly elevated mortality rate (576 deaths per million) (47, 57). Furthermore, some studies observed that countries where older generations had received BCG vaccination exhibited comparatively lower COVID-19 morbidity and mortality (58).

The highest COVID-19 mortality rates were recorded in countries that either never implemented universal BCG vaccination or did so only recently. For instance, Iran introduced universal BCG vaccination in 1984 and exhibited elevated mortality rates, supporting the notion that protection is most pronounced in previously vaccinated elderly cohorts. Countries without universal BCG programs or with discontinued policies—such as San Marino, Belgium, Andorra, Spain, Italy, Sweden, the USA, Saint Maarten, and the Netherlands—rank among those with the highest mortality rates (59).

Multiple epidemiological studies have documented a negative correlation between national BCG vaccination policies and COVID-19 incidence and mortality (60, 61). By the end of 2020, the lowest COVID-19 incidence and mortality rates were observed in countries maintaining mandatory triple-dose BCG vaccination until 2011 (e.g., Belarus, Kazakhstan, Uzbekistan) (62, 63). These findings contradict the null hypothesis of no association between BCG vaccination and COVID-19 outcomes, supporting a potential protective role for BCG (64).

Additionally, epidemiological research has linked BCG vaccination to reductions in respiratory infections, such as respiratory syncytial virus (RSV) and influenza, as well as sepsis in children, with some studies reporting nearly 50% reductions in neonatal mortality in high-risk settings. Timely neonatal BCG administration may significantly improve health outcomes in HIV-1–infected children. Enhanced production of tumor necrosis factor (TNF), interleukins (IL)-1β, IL-6, and interferon-*γ* has been observed in cells from BCG-vaccinated individuals in response to both mycobacterial and heterologous antigens (65, 66).

A double-blind, placebo-controlled phase III trial demonstrated that multi-dose BCG vaccination protects adults with type 1 diabetes against COVID-19 and other infections (67). From April 2021 to November 2022, BCG vaccines derived from the Tokyo strain conferred significant protection against COVID-19 (*p* = 0.023) and robust cross-protection against infectious diseases overall (*p* < 0.0001). Notably, mRNA-based COVID-19 vaccines alone did not demonstrate comparable protection against COVID-19 (*p* = 0.43), and their administration neither enhanced nor interfered with the BCG-dependent protective effect.

In a comparative study of young adults, 11.7% of BCG-vaccinated individuals tested positive for COVID-19 vs. 10.4% of unvaccinated individuals (68). Australian researchers emphasized the continued necessity of BCG vaccination as a safe, effective, and cost-efficient method for tuberculosis prevention, particularly in children, both during and after the COVID-19 pandemic (69).

The pathogenesis of COVID-19 involves hyperinflammation, epithelial barrier dysfunction, and excessive systemic production of inflammatory mediators, especially cytokines. BCG vaccination induces lymphocyte production of INF-γ, which modulates multiple interleukins and may mitigate the severity of COVID-19 by attenuating IL-12 and IL-18–dependent inflammatory responses. Moreover, evidence suggests antigenic cross-reactivity between mycobacterial pathogens and SARS-CoV-2 due to shared peptide sequences and epitope mimicry, implying that adaptive immune responses elicited by BCG vaccination may partially cross-protect against SARS-CoV-2 infection (70, 71).

It remains essential to further investigate the broader immunomodulatory effects of the BCG vaccine without undermining its well-established efficacy in tuberculosis prevention (72, 73). Future studies must rigorously evaluate the safety and effectiveness of BCG vaccination for indications beyond tuberculosis (74).

In the Russian Federation, tuberculosis prevention strategies include the use of both BCG and BCG-M vaccines (75). Both vaccines comply with WHO standards for live attenuated vaccines. While BCG vaccination does not prevent *Mycobacterium tuberculosis* infection *per se*, it effectively protects against severe clinical manifestations, such as miliary tuberculosis and tuberculous meningitis, which result from hematogenous dissemination. Newborns are vaccinated in maternity hospitals between days 3 and 7 of life, with revaccination performed at 6–7 years of age. The Russian Federation enforces strict quality control standards for both BCG and BCG-M vaccines (76).

## 8 HIV infection and BCG vaccination

Children who are exposed to HIV and come into contact with a patients, suffering from tuberculosis, face a significant risk of developing complicated tuberculosis (77). The likelihood of contracting tuberculosis and experiencing severe complications is considerably greater among children with HIV. The BCG vaccine is both safe and effective for infants with HIV, as it prevents severe course of tuberculosis in susceptible children. Early and carefully timed immunization, aligned with traditional vaccination timing and criteria, results in sufficient, moderately strong anti-tuberculosis immunity, as evidenced by local post-vaccination reactions and tuberculin skin tests. The high safety profile of early, cautious BCG vaccination in children with perinatal HIV infection has been established. In contrast, the clinical progression of tuberculosis in unvaccinated children, particularly in younger age groups, tends to be severe and complicated, often leading to rapid deterioration (78).

However, it is important to note that vaccination against tuberculosis in children with HIV does not consistently yield strong immunologic or clinical responses. For example, the Mantoux test (2 TU) is positive in only about one-third of vaccinated HIV-infected individuals. Furthermore, analyses indicate that the incidence of disseminated tuberculosis does not differ significantly between vaccinated and unvaccinated children who are exposed to HIV (79). Children with HIV are at increased risk of disseminated complications, particularly generalized BCG infection, within three years following vaccination.

Currently, children infected with HIV receive vaccinations in accordance with the preventive vaccination schedule (80). Those born to mothers with HIV infection who have undergone three-stage chemoprophylaxis to prevent mother-to-child transmission of HIV are vaccinated against tuberculosis in the maternity hospital (with BCG-M vaccine). Children with confirmed HIV infection using molecular tests for HIV DNA are excluded from BCG vaccination. Vaccination is administered either in the maternity hospital or thereafter, provided there are no clinical or laboratory signs of immunodeficiency (80).

Administration of live vaccines is contraindicated in children with immunodeficiency. Immunization may induce a transient increase in HIV viral replication. Following BCG vaccination, infants with HIV exhibit elevated levels of CCR5+ CD4+ T cells—preferential targets for HIV infection—which can persist for up to eight weeks post-vaccination (43). The risk of HIV acquisition during breastfeeding is also heightened for infants born to mothers with HIV-positive, underscoring the complexity of vaccination decisions in this group.

BCG vaccination in children with HIV involves a delicate balance between benefits and risks, especially concerning vaccine safety, efficacy, and immune response. Infants with HIV are at significant risk for severe BCG-related complications, including disseminated disease, with incidence rates of 329–417 cases per 100,000 vaccinated infants reported in regions of high HIV prevalence (78). Disseminated BCG disease can result in systemic infections involving lungs, bones, or lymph nodes and may carry mortality rates as high as 75%. Additionally, BCG-associated Immune Reconstitution Inflammatory Syndrome (IRIS) frequently occurs after initiation of antiretroviral therapy (ART), presenting as inflammatory lymphadenitis or abscess formation at the vaccination site. Younger age and high baseline viral load are among factors increasing IRIS risk.

Children infected with HIV generally mount suboptimal immune responses to BCG vaccination, characterized by lower levels of protective CD4+ T cells and diminished interferon-gamma production. This inadequate immune response compromises protection against tuberculosis, particularly severe manifestations such as tuberculous meningitis. Consequently, BCG vaccination is not recommended for infants with confirmed HIV infection prior to ART initiation to reduce IRIS risk (68, 81).

Conversely, timely BCG vaccination may confer important non-specific protective effects in infants with HIV. Emerging data provide a foundation for optimizing the timing of BCG vaccination in this population (82). In regions with high tuberculosis incidence, BCG vaccination at birth is recommended when HIV status is unknown, as benefits outweigh risks. Some studies suggest delaying vaccination until HIV status confirmation (e.g., 8–14 weeks) to minimize risks, though this delay may postpone protection against tuberculosis.

BCG vaccination induces immune alterations in infants with HIV, notably increasing activated CCR5+ CD4+ T cells, which could theoretically enhance susceptibility to HIV infection during breastfeeding. However, available primate studies have not demonstrated significant increases in HIV transmission following BCG vaccination (65). These findings highlight the need to carefully balance the timing of BCG vaccination to minimize HIV transmission risk while maximizing vaccine benefits (81).

Several studies suggest that BCG vaccination reduces all-cause mortality in infants with HIV by providing protection against non-tuberculosis infections, such as respiratory viruses, through mechanisms of trained immunity. BCG induces epigenetic and metabolic reprogramming of innate immune cells—including monocytes and macrophages—enhancing their responsiveness to diverse pathogens. This immunomodulatory effect may underlie the observed reductions in mortality among vaccinated infants (83, 84).

## 9 BCG in preterm infants

Children who are exposed to HIV and infected with TB infection face a significant risk of developing complicated tuberculosis (77). The likelihood of contracting tuberculosis and experiencing severe complications is considerably greater among children infected with HIV. The BCG vaccine is both safe and effective for infants with HIV, as it helps prevent the onset of severe tuberculosis in susceptible children. Research indicates that early and carefully timed immunization, aligned with traditional vaccination timing and criteria, results in sufficient, moderately robust anti-tuberculosis immunity, as evidenced by local post-vaccination reactions and tuberculin skin tests. The high safety profile of early, cautious BCG vaccination in children with perinatal HIV infection has been established. In contrast, the clinical progression of tuberculosis in unvaccinated children, particularly in younger age groups, tends to be severe and complicated, often leading to rapid deterioration (78).

It is important to note that vaccination with the TB prevention vaccine in children with HIV does not demonstrate sufficient immunological and clinical efficacy. The Mantoux test with 2TU indicates a positive reaction in only about one-third of vaccinated individuals. An analysis of additional data revealed that the occurrence of disseminated processes in vaccinated patients was not significantly different from that in unvaccinated children exposed to HIV (79). Research has indicated that children with HIV are at an increased risk of developing disseminated complications, particularly generalized BCG infections, within three years post-vaccination. Currently, children infected with HIV receive vaccinations in accordance with the preventive vaccination schedule (80). Those born to mothers with HIV-positive who have undergone three-stage chemoprophylaxis to prevent mother-to-child transmission of HIV are vaccinated against tuberculosis in the maternity hospital (BCG-M). Children who test positive for HIV using molecular methods are excluded from vaccination. BCG vaccination is administered either in the maternity hospital or after the mother and child are discharged, using the BCG-M vaccine, provided there are no clinical or laboratory indications of immunodeficiency (70).

The administration of live vaccines is contraindicated in individuals exhibiting signs of immunodeficiency. Immunization may lead to a temporary rise in HIV viral replication. Research indicates that infants exposed to HIV exhibit an increase in CCR5+ CD4+ T-cell levels following BCG vaccination, with this increase lasting for up to 8 weeks post-vaccination (43). Furthermore, BCG-vaccinated infants aged 8 weeks also show elevated levels of these cells that are preferential targets for HIV. Infants born to mothers with HIV-positive face a heightened risk of HIV infection during breastfeeding, underscoring the importance of vaccination for this vulnerable group. The implications of BCG vaccination in children -infected with HIV involve a complex balance of risks and benefits, particularly concerning vaccine safety, efficacy, and immunological responses. HIV-positive infants are at significant risk for serious complications from BCG, including disseminated disease, with reported incidence rates of 329–417 cases per 100,000 vaccinated infants in regions with high HIV prevalence (78). Such complications can lead to systemic infections affecting the lungs, bones, or lymph nodes, with severe cases carrying a mortality rate as high as 75%. Additionally, Immune Reconstitution Inflammatory Syndrome (IRIS) associated with BCG vaccination is frequently observed after the initiation of antiretroviral therapy (ART), manifesting as inflammatory lymphadenitis or abscesses at the vaccination site. Factors such as younger age and a high baseline viral load increase the risk of IRIS. Children with HIV generally demonstrate suboptimal immune responses to BCG, characterized by lower levels of protective CD4+ T cells and diminished interferon-gamma production. This inadequacy undermines their protection against TB, especially against severe forms such as TB meningitis. Consequently, BCG vaccination is not recommended for confirmed HIV-infected infants prior to starting ART to mitigate the risk of IRIS (68, 81).

At the same time, timely BCG vaccination may have important non-specific protective effects in infants infected with HIV-1. This study may provide a basis for developing optimal timing of BCG vaccination for infants infected with HIV-1 (82). If the HIV status of infants is unknown, BCG vaccination is recommended at birth in areas of high TB incidence, as the benefits outweigh the risks. Some studies suggest delaying BCG until HIV status is confirmed (e.g., at 8–14 weeks) to reduce risks, although this may postpone protection against TB. BCG vaccination increases the number of activated CCR5+ CD4+ T cells (HIV target cells) in HIV-exposed infants, potentially increasing susceptibility to HIV infection during breastfeeding. However, studies in primates have not shown a significant increase in transmission rates (65).

Simultaneously, BCG vaccination triggers immune alterations in infants exposed to HIV, notably increasing the proportion of activated CCR5+ CD4+ cells, which are targets for HIV. These findings provide valuable insights into the balance needed for the timing of BCG vaccine administration, aimed at minimizing the risk of HIV transmission to already infected infants while still providing the potential benefits of the vaccine (81). Several studies indicate that BCG may lower all-cause mortality among infants infected with HIV by offering protection against infections unrelated to tuberculosis (such as respiratory viruses) through trained immunity mechanisms. The BCG is known to induce epigenetic and metabolic changes in innate immune cells, such as monocytes and macrophages, thereby enhancing their response to various pathogens, including respiratory viruses. This could help explain the noted decrease in all-cause mortality among infants who received the vaccination (75, 76).

Using of the BCG vaccine in preterm infants is associated with some concerns due to their immunological immaturity and increased risk of infections. The BCG is generally safe in clinically stable preterm infants (those born at more than 30 weeks' gestation or weighing more than 1.5 kg), with no increased risk of systemic adverse events (e.g., disseminated BCG disease).

A meta-analysis of 10,568 preterm and low birth weight infants found no deaths or systemic reactions associated with BCG vaccination within 7 days after birth. Forty studies were included in the meta-analysis, involving preterm infants (born at 26–37 weeks of gestational age) and/or small-for-date infants (0.69–2.5 kg at birth). Based on the available data, early BCG vaccination of healthy preterm infants and/or children with low birth weights in order to improve vaccine efficacy is justified. Local reactions (e.g., lymphadenitis, ulceration) occurred with the same frequency (0%–4.2%) as reported in term infants (85).

Extremely preterm infants (<30 weeks or <1.5 kg) and those with immunodeficiency (e.g., SCID, HIV) face higher risks of disseminated BCG infection, which can be fatal (86). In China, severe adverse events (e.g., interstitial pneumonia, and sepsis) were rare (8 per million) but had a 100% mortality rate in preterm infants (87).

Preterm infants mount cell-mediated immune responses (e.g., tuberculin skin test conversion, lymphocyte proliferation) similar to term infants when vaccinated at 34–40 weeks' postconceptional age. No significant differences in scar formation or cytokine profiles were observed between early (34–35 weeks) and late (38–40 weeks) vaccination terms (88).

BCG may enhance trained immunity, reducing all-cause mortality and respiratory infections in preterm infants. It is recommended for stable preterm infants (>30 weeks, >1.5 kg) to improve coverage and leverage potential non-specific immune benefits. Delayed vaccination increases dropout rates in high-TB-burden settings. Vaccination is advised to be postponed only for extremely preterm infants or those with comorbidities, until they reach approximately 34 weeks' postconceptional age or achieve clinical stability.

WHO supports BCG vaccination for preterm infants in high-TB-burden regions but emphasizes the importance of personalized risk assessment (89).

## 10 BCG complications in primary immunodeficiencies

The development of complications after BCG vaccination may indicate the presence of primary immunodeficiency. It is particularly important to be aware of this fact if there is a family history of BCG complications, immunodeficiency, or unexplained deaths of children following vaccination.

Immunological screening of patients with suspected immunodeficiency is relevant, as it can help prevent the development of severe complications after vaccine prophylaxis and reduce morbidity and mortality associated with BCG vaccination (90, 91). The most severe complications associated with BCG vaccination in the context of primary immunodeficiency include: generalized BCG infection, BCG osteitis (osteomyelitis), and disseminated BCG lymphadenitis (92, 93).

**Figure 8:**
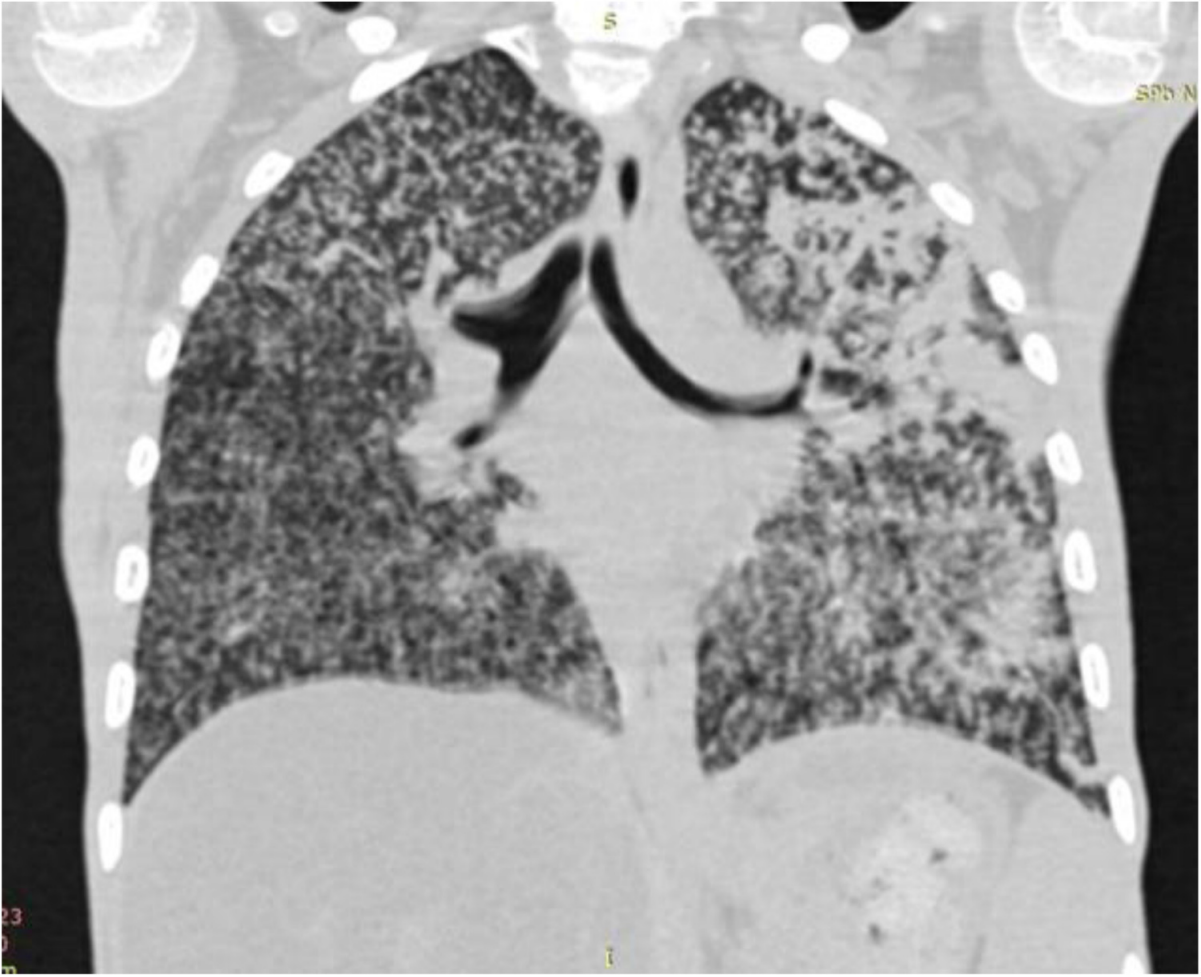
X-ray of a child with зulmonary myliary dissemination BCG infection.

Generalized BCG infection is the most severe complication resulting from dissemination of BCG *Mycobacteria* throughout the body. Fever, weight loss, hepatosplenomegaly, and involvement of lymph nodes, skin, and lungs are common clinical features of that entity. Lethality exceeds 50%. It is more common in severe combined immunodeficiency (SCID) and chronic granulomatous disease. Immunocompromised patients with generalized BCG infection may present with sole axillary lymphadenopathy in 64%, combined axillary, cervical or supraclavicular lymphadenopathy in 32%, hepatomegaly and splenomegaly in 50%, pneumonia in 36%, gastrointestinal symptoms in 10%, skin rash in 15%, meningitis in 5%, and osteomyelitis in 1% (90) (Figure 8).

**Figure 9:**
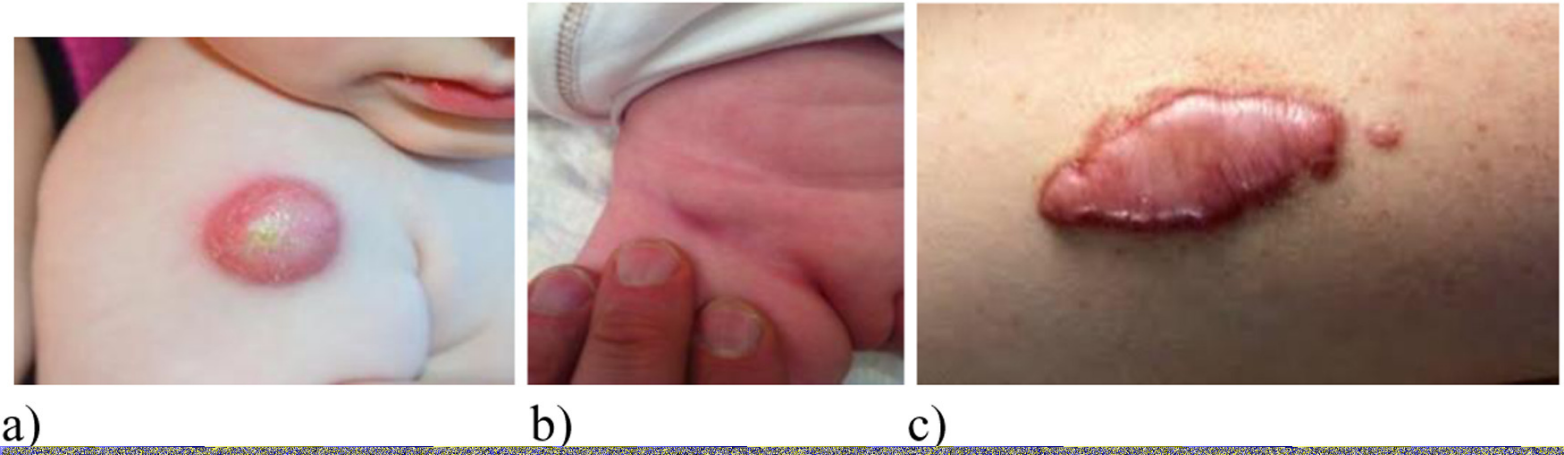
Variants of local manifestations of complicated course of BCG vaccination **(A)** cold abscess, **(B)** inguinal lymphadenitis after BCG vaccination in the buttock (Sweden); **(C)** keloid scar after vaccination).

BCG dissemination can occur in 65% with a fatality rate of 36%. Delaying BCG vaccination until 6 months of age significantly reduces the incidence of BCG-related complications in patients suffering from SCID. It is the importance of developing individualized vaccination schedules for high-risk groups (Figure 9). Early newborn screening and timely diagnosis of immunodeficiencies are essential to further reduce complication rates (94, 95).

Localized reactions such as cold abscess, ulcers, keloid scars may also occur (84).

**Figure 10:**
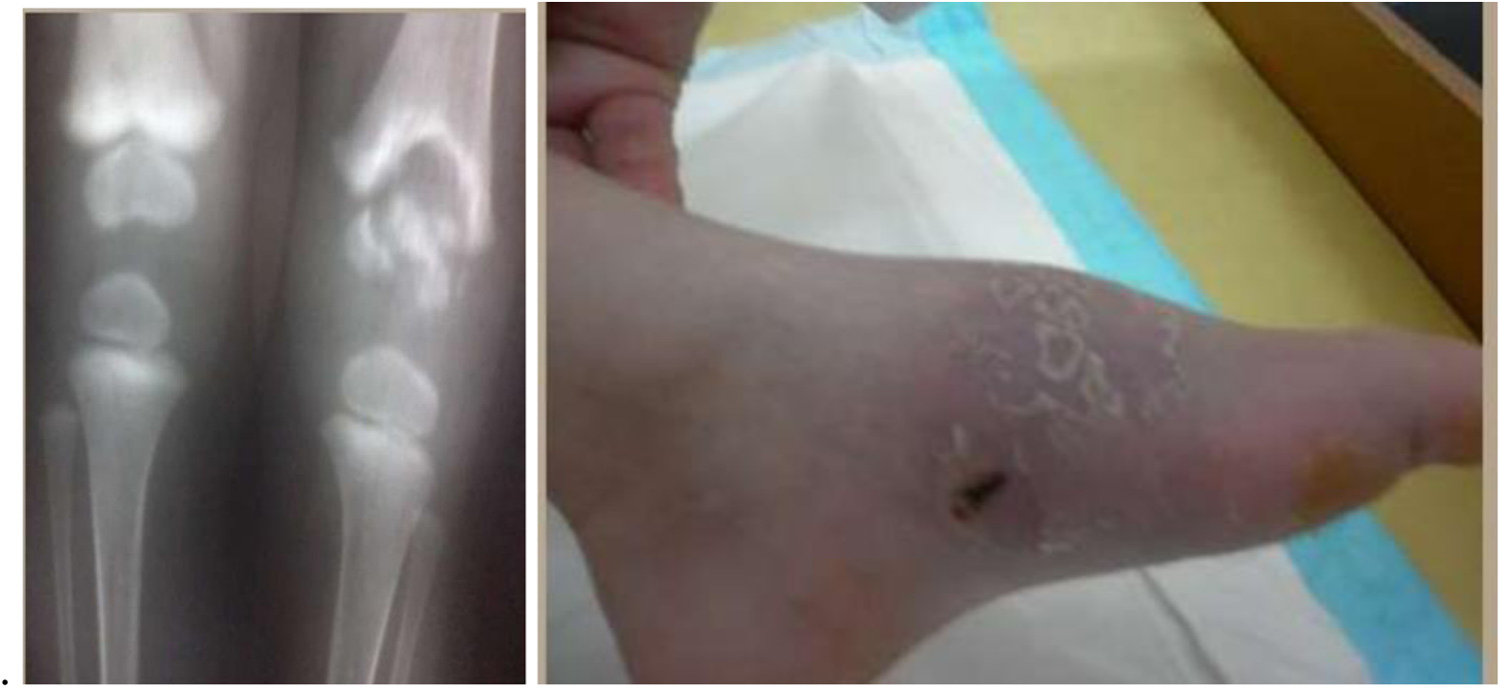
BCG ostiomyelitis in a 4-year-old child (radiographs and tibial fistula). BCG bovis DNA was obtained.

BCG osteitis (osteomyelitis) occurs in bones and joints, developing several months or even years after vaccination. Most often metaphyses of long tubular bones, vertebrae, and sternum are affected. This complication is associated with defects in the IL-12/IFN-*γ* pathway leading to impaired immune response against BCG *Mycobacteria* (96, 97) (Figure 10).

Disseminated BCG lymphadenitis can occur not only in regional (axillary an/or cervical) lymph nodes, but also in their distant groups. It may be accompanied by suppuration, fistula formation. It often occurs in HIV infection and combined immunodeficiencies (90).

## 11 Latest advances in vaccine strategies and noninvasive imaging methods for tuberculosis diagnosis and monitoring

The development of new vaccines remains a strategically important and urgent task in the global fight against tuberculosis (TB). In 2023, the WHO Council for Accelerating the Development of a Tuberculosis Vaccine was established to expedite vaccine development by leveraging lessons learned from the COVID-19 pandemic (98, 99). Several vaccine candidates, including RUTI and DAR-901, are currently being evaluated as immunotherapies aimed at shortening treatment duration and preventing relapse, particularly in cases of drug-resistant TB. Novel platforms such as mRNA-based and viral vector vaccines show promising early results. For instance, mRNA vaccines like BNT164 (BioNTech) and viral vector vaccines such as ChAdOx1-85A are in early clinical trials designed to elicit strong T-cell immune responses (85, 99, 100).

A notable example is the mRNACV2 vaccine, an mRNA-based subunit vaccine formulated with lipid nanoparticles (LNP) and an adjuvant. In preclinical studies, intramuscular administration of mRNACV2 to female C57BL/6 mice—either as a standalone vaccine or as a booster following BCG vaccination—induced a high frequency of multifunctional, antigen-specific Th1 CD4+ T cells in blood and lungs. This immune activation was associated with rapid recruitment of both innate and adaptive immune cells to draining lymph nodes. Importantly, mRNACV2 vaccination conferred significant lung protection in mice infected *M. tuberculosis*- by reducing bacterial load and inflammatory infiltration. When used as a booster, mRNACV2 enhanced immune responses and provided durable protection in BCG-vaccinated mice (100, 101). It is also known that circRNA vaccines represent a potential new direction in the vaccine era. Several circRNA vaccines have recently been synthesized and tested *in vitro* and *in vivo* (98).

In several publications, researchers have described the development of a lipid nanoparticle (LNP)–mRNA-based vaccine, referred to as mRNACV2, which encodes the *Mycobacterium tuberculosis* fusion protein CysVac2. Previously, this vaccine was classified as an adjuvanted subunit vaccine. The LNP–mRNA vaccine was administered intramuscularly to female C57BL/6 mice either as a standalone immunization or as a booster following BCG vaccination, to assess the immunogenicity and efficacy of the construct. Notably, mRNACV2 enhanced immune responses and provided long-term protection when used as a booster in BCG-vaccinated mice. The findings underscore the potential of the LNP–mRNA platform for tuberculosis control and support further research to facilitate its translation to human use (99).

Challenges in TB vaccine development and implementation include variable vaccine efficacy across different population groups and regions. The inconsistent protection offered by BCG highlights the need for new vaccines that confer effective immunity across all age groups and geographic areas. Funding shortages pose a significant barrier; currently, only 26% of the estimated $22 billion required annually for TB control programs is available, risking delays in vaccine rollout (88, 89).

In parallel with vaccine development, there is a critical need to advance non-invasive imaging technologies for TB diagnosis and treatment monitoring (101, 102). Molecular imaging using SPECT/CT targeting the translocator protein (TSPO) with radioligands such as [^125I]iodo-DPA-713 has shown high specificity for TB-associated inflammation in macrophages, with superior signal-to-noise ratios compared to conventional [^18F]FDG-PET. This enables real-time 3D visualization of TB lesions, overcoming limitations of sputum-based diagnostics, which cannot detect non-respiratory tract lesions (102). Furthermore, novel PET/CT radiopharmaceuticals like ^68Ga-labelled somatostatin analogues are being introduced to improve specificity over [^18F]FDG, which may be confounded by non-TB inflammation. PET/CT enables longitudinal assessment of treatment response and drug penetration into granulomas, critical parameters for evaluating novel therapies (103). However, these imaging modalities are limited by high costs and lack of pathogen-specificity, restricting their use in resource-limited settings.

Near-infrared (NIR) spectroscopy using semiconductor sensors represents a promising, low-cost diagnostic alternative. Diluted III-V semiconductors, such as nitrogen-doped GaAs, enhance NIR sensitivity to detect TB biomarkers in sputum or breath samples rapidly and in mobile formats, potentially addressing diagnostic gaps in low-resource environments (104). Additionally, fluorescence labeling with iron transport protein (IrtAB) enables detection of M. tuberculosis in saliva within 10 min, considerably faster than culture-based methods.

## 12 Conclusion

The BCG vaccine has a well-established history of development and use spanning more than a century. Its widespread administration has contributed significantly to reducing childhood mortality from tuberculosis worldwide (105, 106). In the Russian Federation, approximately 90% of newborns receive the BCG vaccine, and as a result**,** the incidence of disseminated tuberculosis in young children remains low.

Ongoing research has revealed that BCG strains have genetically diverged from the original strain, resulting in notable genomic differences. Although these variations have been associated with changes in scar formation, reactogenicity, and immune response profiles, their impact on overall vaccine efficacy remains inconclusive (105, 107,108,109,110,111).

The BCG vaccine has demonstrated the capacity to induce mucosal and systemic immune responses, offering partial protection not only against tuberculosis but also against other infections involving mucosal surfaces, such as salmonellosis, certain viral respiratory infections, and possibly HIV. Through mechanisms of trained immunity, early BCG vaccination may enhance resistance to malaria and lower the incidence of lymphoid malignancies. It is also used intravesically as an immunotherapy for bladder cancer. While it is important to explore the broader immunomodulatory potential of BCG, such efforts should not overshadow its established efficacy in preventing severe tuberculosis (93, 98).

Although BCG vaccination does not eliminate infection with *Mycobacterium tuberculosis*, it effectively prevents severe disease manifestations such as miliary tuberculosis and tuberculous meningitis, which are associated with hematogenous dissemination. To minimize the risk of serious complications, including generalized BCG infection, neonatal screening for primary immunodeficiencies should be considered before vaccine administration.

Some restrictions remain on the use of BCG in preterm infants due to concerns regarding their immature immune systems and increased susceptibility to infections. Nevertheless, the vaccine is generally considered safe for clinically stable preterm infants born after 30 weeks of gestation or weighing more than 1.5 kg.

A persistent challenge involves protecting children with HIV-infected and tuberculosis while minimizing the risk of vaccine-related adverse events in those with underlying immunodeficiencies. In addition, the development of new vaccines effective against drug-resistant *M. tuberculosis* strains, and suitable for use across different age groups and populations, remains a critical priority.

In summary, the BCG vaccine remains one of the most essential tools in global tuberculosis prevention. Beyond its role in TB control, it contributes to the modulation of immune reactivity through its adjuvant properties, making it a subject of continued scientific interest.
